# Magnetic Effect on the Performance of a Four-Frequency Differential Laser Gyroscope

**DOI:** 10.3390/s26061927

**Published:** 2026-03-19

**Authors:** Guochen Wang, Jiaqi Li

**Affiliations:** College of Advanced Interdisciplinary Studies, National University of Defense Technology, Changsha 410073, China

**Keywords:** four-frequency differential ring laser gyroscope, magnetic effect, plasma movement, Lorentz force, Zeeman effect, frequency stabilization optimal operating point

## Abstract

The performance of a four-frequency differential laser gyroscope (FFDLG) is severely affected by the magnetic field. In this paper, the following conclusions are discussed through theoretical analyses and experimental data: First of all, the Zeeman effect cannot fully explain the magnetic effect on the gain region due to the plasma movement. Secondly, an FFDLG does not have a unique optimal operating point where the gyroscope is not affected by any magnetic field. Plasma movement driven by Lorentz force induces a Fresnel drag effect, leading to a frequency imbalance and magnetic error in the ring laser gyroscope (RLG). This mechanism, involving the interaction between moving gain media and the counter-propagating beams, was missed in previous research.

## 1. Introduction

The Four-Frequency Differential Laser Gyroscope (FFDLG) is a high-precision inertial sensor widely utilized in orientation, navigation, guidance, and control systems [1,2,3]. Compared to traditional mechanical gyroscopes and fiber optic gyroscopes, the FFDLG offers significant advantages, including the absence of mechanical noise, a solid-state architecture, high scale-factor stability, and zero delay [4]. Recent reviews emphasize that despite the emergence of new sensor technologies, Ring Laser Gyroscopes (RLGs) remain indispensable for high-end navigation and strategic defense applications due to their superior long-term stability and precision [5].

However, the performance of the FFDLG is severely limited by its sensitivity to environmental factors, particularly magnetic fields. Early studies in the United States and Europe established the foundational understanding of how magnetic fields interact with the gain medium to produce bias errors [1,2,3]. Building on this foundation, more recent research has focused on refining error models. For instance, Azarova et al. provided a comprehensive review of the optical schemes and operation principles of modern ZLGs, highlighting the critical role of frequency biasing [6]. Similarly, Kolbas et al. investigated the effects of both external and internal magnetic fields on bias stability, distinguishing between magnetic and non-magnetic components of zero bias [7].

Current research continues to address these challenges with a focus on multi-physics error coupling. Recent analyses have explicitly estimated the influence of external magnetic fields on error budgets in nonplanar symmetric cavities [8]. Furthermore, studies in 2025 expanded the scope of error analysis by investigating the coupling between cavity thermal deformations and active path length stabilization, demonstrating that environmental stability remains a key bottleneck [9,10]. While compensation methods for magnetic sensitivity have been proposed to address these complex interactions [11], even advanced large-scale ring laser projects have confirmed that magnetic noise remains a fundamental limit to achieving ultimate sensitivity [12].

Despite this progress, most existing studies [6,7,11] have analyzed the magnetic error primarily through the lens of the Zeeman effect—where the magnetic field splits atomic energy levels. These analyses often overlook the dynamic interaction between the magnetic field and the plasma itself. This paper addresses this gap by investigating a physical mechanism that has been largely neglected: the effect of the Lorentz force on plasma movement within the gain region. We hypothesize that the Zeeman effect alone cannot fully explain the observed magnetic sensitivity, particularly the complex behavior of the “optimal operating point.” Through theoretical analysis and experimental verification, we demonstrate that plasma movement induced by the Lorentz force contributes significantly to the magnetic error. Furthermore, we show that contrary to the conclusions of some earlier studies, an FFDLG does not possess a unique, universal optimal operating point that is immune to all magnetic field frequencies and amplitudes.

## 2. Theoretical Principle

### 2.1. Principle of FFDLG

**Figure 1:**
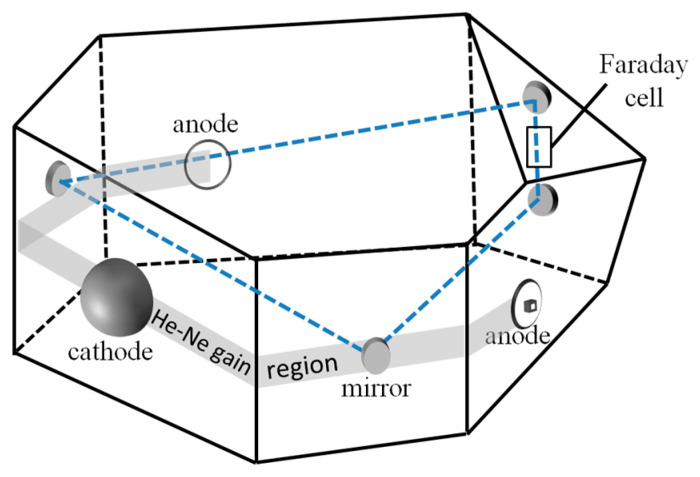
Schematic diagram of a non-planar four-frequency differential laser gyroscope (NFFDLG).

The schematic diagram of the non-planar FFDLG (NFFDLG) used in the experiment is illustrated in Figure 1, in which the optical path formed by four mirrors is non-planar. Two anodes and a common cathode constitute two symmetrical discharge paths, which are connected to a high-voltage DC steady-current power supply to provide gain for He-Ne gas through discharge. The power supply can provide two currents of 10^−4^ degree of symmetry, and the influence of Langmuir flow on gyro bias is eliminated by the offset effect of two-way gas flow.

**Figure 2:**
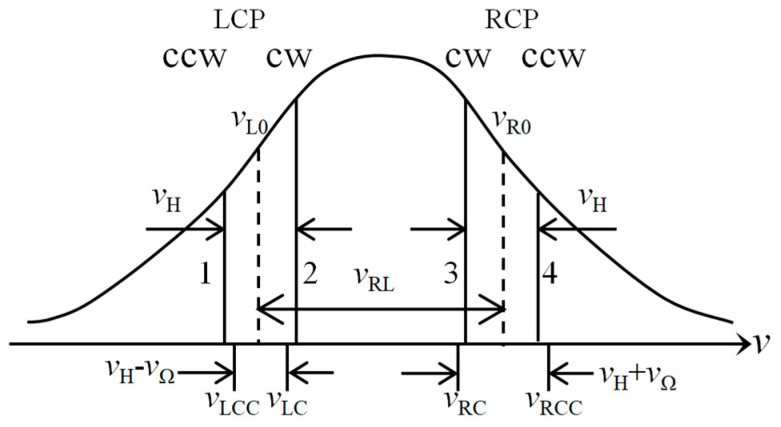
Mode spectrum of an NFFDLG.

Owing to the frequency-splitting effect induced by the non-planar path and the effect of the Faraday rotator passive element(also called the Faraday cell), four distinct frequency modes are established within the resonant cavity: left-circularly polarized beam propagating in the clockwise direction ($v_{LC}$), left-circularly polarized beam propagating in the counter-clockwise direction ($v_{LCC}$), right-circularly polarized beam propagating in the clockwise direction ($v_{RC}$) and right-circularly polarized beam propagating in the counter-clockwise direction ($v_{RCC}$).The corresponding mode spectrum of the NFFDLG is illustrated in Figure 2.

The frequency difference $v_{RL}=v_{R0}-v_{L0}$ between the center frequencies of the left-circularly polarized (LCP)mode pair and right-circularly polarized (RCP)mode pair is very large, often reaching several hundred MHz. Therefore, the two LCP modes form a LCP gyroscope, and the two RCP modes form a RCP gyroscope. For an input angular velocity Ω, the beat frequencies of LCP and RCP resonant modes are expressed as:

(1) $$\begin{array}{l} v_{L}\equiv v_{LC}-v_{LCC}=v_{H}-v_{\Omega }\\ v_{R}\equiv v_{RCC}-v_{RC}=v_{H}+v_{\Omega } \end{array}$$

where $v_{L}$ is the beat frequency of the LCP mode pair, $v_{R}$ is the beat frequency of RCP mode pair, $v_{H}$ is the Faraday offset frequency, and $v_{\Omega }$ is the Sagnac frequency shift induced by the input angular velocity Ω. The difference and the sum of the output signal $v_{L}$ and $v_{R}$:

(2) $$\begin{array}{l} v_{O}=v_{R}-v_{L}=2v_{\Omega }=\frac{8A}{\lambda L}\cdot \Omega \\ v_{SUM}=v_{R}+v_{L}=2v_{H} \end{array}$$

where $v_{O}$ is the difference between $v_{L}$ and $v_{R}$, which is proportional to the input angular velocity Ω. The term of $v_{SUM}$ is two times the Faraday offset frequency $v_{H}$. Additionally, *A* is the area enclosed by the optical path, *L* is the geometrical length, and λ is the wavelength. Due to the differential effect, the stability of $v_{H}$ does not affect $v_{O}$. Furthermore, the scale factor of an NFFDLG is twice that of a single gyroscope with two modes, thus the sensitivity of an NFFDLG is doubled.

The NFFDLG has two different working states: one is when the LCP modes are in the low-frequency region and the RCP modes are in the high-frequency region, such as in Figure 2; the other state is when the RCP modes are in the low-frequency region and the LCP modes are in the high-frequency region. In practice, the state at which the NFFDLG works is decided by the frequency stabilization circuit. The two states have no essential difference. Therefore, it is only need to ensure the FFDLG operates in the same state every time.

### 2.2. Theoretical Principles of FFDLG and the Sources of Magnetic Effect in FFDLG

If the second type of lock-in effect in FFDLGs is disregarded, the study of FFDLG can proceed within the theoretical framework of the nearly independent gyroscope approximation. This simplification is valid for investigating FFDLG performance in most practical scenarios because the primary errors in FFDLGs are caused by coupling effects within each individual gyroscope. Coupling between different gyroscopes is significantly weaker. All subsequent discussions in this paper are based on nearly independent gyroscope approximation.

With Ref. [13] and Figure 2, the output frequency of an FFDLG is given by the following equation:

(3) $$\begin{array}{lll} 2\pi \times v_{O} & = & \omega_{O}=\left(\omega_{2}-\omega_{1}\right)-\left(\omega_{4}-\omega_{3}\right)\\ & = & \left(1+\mathrm{SFC}\right)\times \left(2\omega_{\Omega }\right)+\left({\mathrm{SFC}}_{12}{-\mathrm{SFC}}_{34}\right)\times \left(\omega_{H}+\omega_{B}\right)-\mathrm{SFC}\times 4\mathrm{KV}\\ & & -\left[\frac{\rho \left(\xi_{12}\right)-\tau \left(\xi_{12}\right)}{\beta \left(\xi_{12}\right)-\theta \left(\xi_{12}\right)}\times \left(\gamma_{2}-\gamma_{1}\right)-\frac{\rho \left(\xi_{34}\right)-\tau \left(\xi_{34}\right)}{\beta \left(\xi_{34}\right)-\theta \left(\xi_{34}\right)}\times \left(\gamma_{4}-\gamma_{3}\right)\right] \end{array}$$

In Equation (3), $\omega_{1}=2\pi \cdot v_{LCC}$, $\omega_{2}=2\pi \cdot v_{LC}$, $\omega_{3}=2\pi \cdot v_{RC}$, $\omega_{4}=2\pi \cdot v_{RCC}$, $\omega_{\Omega }=2\pi \cdot v_{\Omega }$, $\omega_{H}=2\pi \cdot v_{H}$, $\omega_{B}=2\pi \cdot v_{B}$, and $v_{B}$ is the Zeeman splitting frequency induced by a longitudinal magnetic field *B*; ${\mathrm{SFC}}_{12}$ and ${\mathrm{SFC}}_{34}$ are the relative scale factor corrections for the LCP and RCP beams, respectively, both of which are induced by the anomalous dispersion of the gain medium; SFC is the relative scale factor correction of the FFDLG, and $\mathrm{SFC}=\frac{1}{2}\left({\mathrm{SFC}}_{12}+{\mathrm{SFC}}_{34}\right)$; $K=\frac{2\pi }{\lambda }$ is the wave number, defined as 2π times the number of wavelengths per unit length; V is the average Langmuir flow velocity in the discharge gain regions; ρ, τ, β, θ are the self-repulsion coefficient, mutual-repulsion coefficient, self-saturation coefficient, and cross-saturation coefficient, respectively. γ denotes the round-trip loss; $\xi_{12}=\frac{\xi_{1}+\xi_{2}}{2}$ and $\xi_{34}=\frac{\xi_{3}+\xi_{4}}{2}$ are the induced frequency parameters, and $\xi_{j}=\frac{\omega_{j}-\omega_{0}}{\mathrm{Ku}},\;j=1,2,3,4$; ($\frac{\mathrm{Ku}}{2\pi }\sqrt{\log2}$ is the half width of the Doppler bandwidth, and $\frac{\omega_{0}}{2\pi }$ is the center frequency of positive and negative rotational optical gain curve); −SFC×4KV is the Langmuir-flow-induced error term, and $-\left[\frac{\rho \left(\xi_{12}\right)-\tau \left(\xi_{12}\right)}{\beta \left(\xi_{12}\right)-\theta \left(\xi_{12}\right)}\times \left(\gamma_{2}-\gamma_{1}\right)-\frac{\rho \left(\xi_{34}\right)-\tau \left(\xi_{34}\right)}{\beta \left(\xi_{34}\right)-\theta \left(\xi_{34}\right)}\times \left(\gamma_{4}-\gamma_{3}\right)\right]$ is the difference loss term. For an FFDLG, the following relations hold: $\gamma_{4}=\gamma_{1}$, $\gamma_{3}=\gamma_{2}$.

According to the material, size and shape of the Faraday cell, the offset frequency can be obtained by:

(4) $$\begin{array}{l} \nu_{H}/B\approx 10\mathrm{MHz}/\mathrm{Tesla}\\ \nu_{H}=\omega_{H}/2\pi \end{array}$$

where *B* is the magnetic flux density. According to Ref. [14] and Equation (3), $\left({\mathrm{SFC}}_{12}-{\mathrm{SFC}}_{34}\right)$ is about $10^{-6}$, the error can be estimated as:

(5) $$\left|\nu_{H}\left({\mathrm{SFC}}_{12}-{\mathrm{SFC}}_{34}\right)\right|/B\approx 10\mathrm{Hz}/\mathrm{Tesla}$$

If the magnetic field changes $10^{-4}$ Tesla (i.e., 1Gauss), the resulting drift would change 0.001 Hz due to the Faraday cell.

According to Ref. [5] and Equation (3), $\nu_{B}$ is about $\nu_{B}/B\approx 35\mathrm{GHz}/\mathrm{Tesla}$, so longitudinal Zeeman effect error is estimated by:

(6) $$\left|\nu_{B}\left({\mathrm{SFC}}_{12}-{\mathrm{SFC}}_{34}\right)\right|/B\approx 35\mathrm{KHz}/\mathrm{Tesla}$$

If the magnetic field changes $10^{-4}$ Tesla (1Gauss), the drift would change 0.0035 Hz due to the longitudinal Zeeman effect.

From Equations (5) and (6), it can be concluded that the error induced by Faraday effects is significantly smaller than that induced by longitudinal Zeeman effects. Therefore, in practical applications, efforts should be focused on restraining longitudinal Zeeman effect errors.

### 2.3. Optimal Operating Point Control and Dispersion Balance Control

To enhance FFDLG’s performance, two control schemes optimal operating point control and dispersion balance control—are proposed in Ref. [3]. These schemes are theoretically derived from the error term $\left({\mathrm{SFC}}_{12}-{\mathrm{SFC}}_{34}\right)\times \left(\omega_{H}+\omega_{B}\right)$ in Equation (3). In optimal operating point control, the frequency stabilization circuit works at a specific point where the error term satisfies $\left({\mathrm{SFC}}_{12}-{\mathrm{SFC}}_{34}\right)=0$, so that the performance of the FFDLG is insensitive to magnetic fields. Dispersion balance control involves adjusting the magnetic field so that the error term satisfies $\left(\omega_{H}+\omega_{B}\right)=0$, so that the FFDLG performance is not affected by the operating point of the frequency stabilization. The performance of an FFDLG is intensively affected by the magnetic field. Therefore, the optimal operating point control is a practical solution if it can reduce the magnetic error effectively in practical applications. Although dispersion balance control can relax the precision requirements of frequency stabilization by applying an appropriate magnetic field on the gain region, achieving both control conditions $\left({\mathrm{SFC}}_{12}-{\mathrm{SFC}}_{34}\right)=0$ and $\left(\omega_{H}+\omega_{B}\right)=0$ simultaneously is challenging. This difficulty arises from angle random walk, which makes it nearly impossible for both schemes to reach the necessary precision concurrently. Fortunately, current frequency stabilization technology offers sufficiently high precision, rendering dispersion balance control unnecessary in many practical scenarios.

While both schemes have been investigated in detail in Refs. [15,16,17,18], this paper focuses primarily on optimal operating point control.

## 3. Experiment Scheme

To dynamically adjust the frequency stabilization operating point, a variable magnetic field should be imposed as the error excitation source. When the output of the FFDLG does not vary with the variation in the magnetic field, the optimal operating point is found. Although Ref. [3] mentions the optimal operating point control and dispersion balance control, it does not discuss how to realize the two control schemes. The Refs. [16,17] discuss the implementation of adaptive path length control and multioscillator stabilization techniques. The Ref. [18] establishes the fundamental theoretical basis for operating point selection.

### 3.1. Generation of the High-Frequency Alternating Magnetic Field

**Figure 3:**
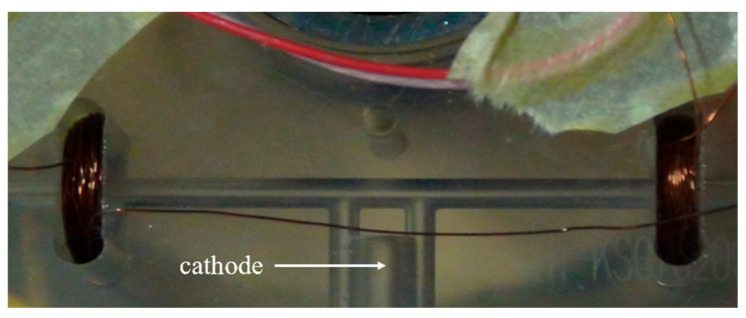
Coils in the gain region of the FFDLG.

To generate the required magnetic field for the optimal operating point control, two holes were symmetrically drilled on both sides of the gyroscope cathode. Coils were wound inside these holes and then connected. The coils within the gain region are illustrated in Figure 3. During the high-level of the control signal, a positive current flows through the coils, while a negative current flows during the low-level period. This configuration produces the required high-frequency alternating magnetic field.

**Figure 4:**
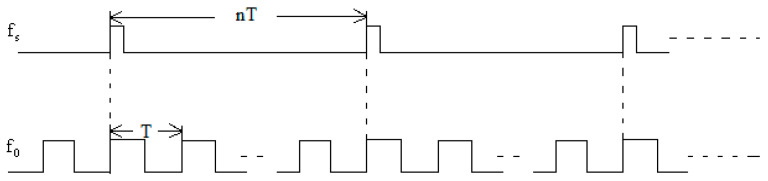
Sequence relationship between the high frequency magnetic field and the sampling signal of FFDLG.

The sampling frequency of gyroscope output signal is denoted by $f_{S}=1/T_{S}$. To eliminate the influence of the applied magnetic field on the gyroscope output, the driving high-frequency alternating current is configured as a square wave with equal positive and negative amplitudes. Its frequency $f_{0}=1/T$ satisfy $f_{0}=n\times f_{S}$, where n is a positive integer (equivalently, $T_{S}=n\times T$). Ideally, the amplitude of the generated magnetic field is proportional to the coil current amplitude, and its frequency coincides with that of the current. Therefore, the temporal relationship between high frequency alternating magnetic field and the sampling signal of FFDLG must strictly follow the timing sequence shown in Figure 4. Each sampling period $T_{s}$ contains n complete cycles of the magnetic field period T. Within each magnetic field cycle, the positive and negative magnetic fields are of equal duration and magnitude. Consequently, the cumulative effect of the magnetic fields on the gyroscope output over one sampling period $T_{s}$ is zero, ensuring the gyroscope output remains affected by the applied magnetic field. During the experiment, no visible changes in plasma brightness or discharge intensity were observed when the alternating current was applied to the coils. This confirms that the coil current (0.016 A to 0.215 A) is sufficiently small to avoid interfering with the DC discharge balance.

### 3.2. Calculation for Error Induced by Magnetic Field

The error induced by the magnetic field is defined as the difference between the gyroscope output under a positive magnetic field and that under a negative magnetic field. To mitigate the effects of bias instability and external disturbances (such as motion) on the error calculation, an overlapping sliding-window weighting algorithm is employed. This method accumulates error data over multiple consecutive magnetic cycles, typically requiring an integration time of at least 10 s. Assuming the alternating magnetic field switches 2*N* times within an operating point adjusting period, the gyroscope output captured by the counting circuit are sequenced as follows:

(7) $$\begin{array}{l} \mathrm{magnetiv}\;\mathrm{field}\;(+):\;1\;3\;\cdot \cdot \cdot \cdot \cdot \;2N-1\\ \mathrm{magnetiv}\;\mathrm{field}\;(-):\;\;2\;4\;\cdot \cdot \cdot \cdot \cdot \cdot \cdot \cdot \cdot \cdot \cdot \cdot 2N \end{array}$$

The individual error is extracted using a weighted sliding-window algorithm:

(8) $$e_{k}=0.5\cdot x_{k}+x_{k+1}+0.5\cdot x_{k+2}\;(k=1,2,\ldots ,2N-2)$$

The final bias error *E* induced by the alternating magnetic field during the adjustment of the frequency stabilization operating point is defined as the cumulative mean of the individual calculation results:

(9) $$E=\frac{1}{M}\sum_{k=1}^{2N-2}{\left(-1\right)}^{k-1}\cdot e_{k}$$

The error is extracted using a weighted sliding-window algorithm. It is important to note that since the magnetic field is modulated as a high-frequency square wave and the error is calculated as the difference between the positive and negative states (Equation(8)), any static system bias or slow-drifting bias is naturally canceled out. Therefore, the “Average Error” presented in the results represents the specific sensitivity of the gyro to the magnetic field (Lorentz and Zeeman effects) rather than a shift in the underlying system bias. Furthermore, the intrinsic bias of the FFDLG used in this study is approximately 0.005°/h in the absence of an external magnetic field. Since the magnetic-induced errors observed in our experiments are orders of magnitude larger than this baseline stability, the influence of the intrinsic system bias is negligible and does not interfere with the identification of the magnetic effects.

### 3.3. Feedback Control Logic for the Operating Point

The bias error *E*, derived in Section 3.2, serves as the feedback signal for the frequency stabilization system. The “operating point” is physically defined by the longitudinal mode position relative to the gain curve, which is monitored via the light intensity difference between the LCP and RCP beams. When a non-zero error E is detected under the alternating magnetic field, the frequency stabilization circuit applies a DC offset to the path-length control voltage (via the piezoelectric transducer). This adjustment process continues until the magnetic-induced error *E* is minimized, effectively locking the gyroscope at its optimal operating point for the given magnetic environment.

### 3.4. Iterative Calibration and Long-Term Maintenance

To ensure the FFDLG remains at its highest precision, the process of identifying the optimal operating point (described in Section 3.2 and Section 3.3) is performed iteratively. This is necessary because environmental factors, such as temperature fluctuations and internal stress relaxation, can cause a slow drift in the position of the optimal point over time [13]. For long-term navigation applications, the frequency stabilization circuit is designed to re-calibrate and update the operating point at regular intervals, ensuring that the magnetic sensitivity remains minimized throughout the mission.

## 4. Experiment Verification

There are discussions and conclusions on the optimal operating point for frequency stabilization in Refs. [15,16,17,18]. This paper will verify whether that point for an FFDLG is invariant to changes in the frequency and amplitude of the magnetic fields. Verifying this universal is crucial, as directly determines the practical value of this approach.

The experimental procedure is as follows:

1. The gyroscope was preheated for over 2 h. The error calculation and adjustment cycle for frequency stabilization operating point was set to 25 s;
2. Two magnetic fields with different amplitudes were applied. For each amplitude, the frequency was varied across seven levels to identify the corresponding error induced by magnetic field and optimal operating point;
3. For each condition, seven experimental runs were performed. In the first six runs, the frequency stabilization circuit was set the position that light intensity voltage difference between RCP and LCP beams was zero. The errors induced by magnetic field from these runs were recorded and averaged to obtain the average error. In the seventh run, the operating point was adjusted to identify the optimal operating point, the position where the magnetic field did not affect the FFDLG output. The position of the optimal operating point defined as proportion between the light intensity voltage difference of RCP and LCP and the light intensity voltage sum of RCP and LCP, and was recorded in percentage;
4. The gyroscope remained powered throughout the entire process to eliminate error induced by repeated restarts;
5. An additional test was conducted under a low-amplitude magnetic field to evaluate the optimal operating point at frequencies of 0.04 Hz and 4 KHz.

**Figure 5:**
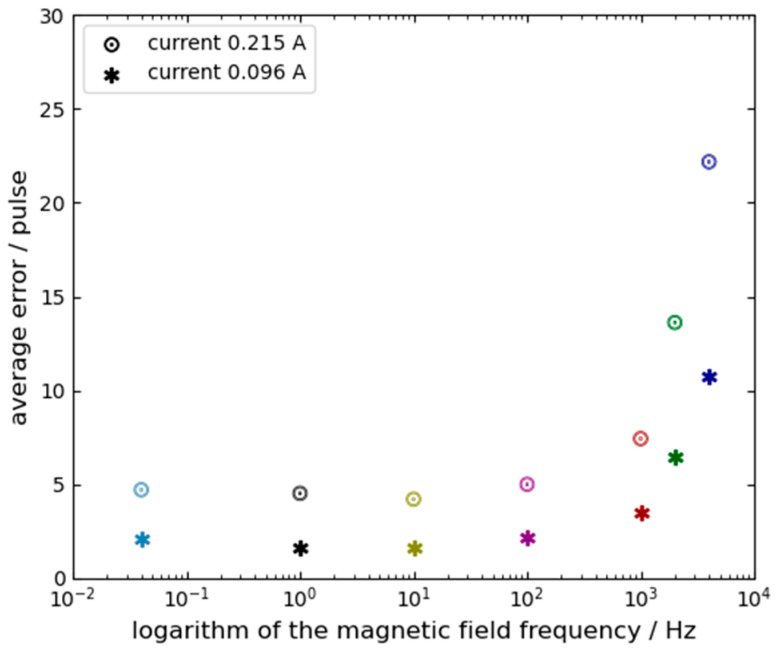
Average magnetic-induced error versus magnetic field frequency.

**Figure 6:**
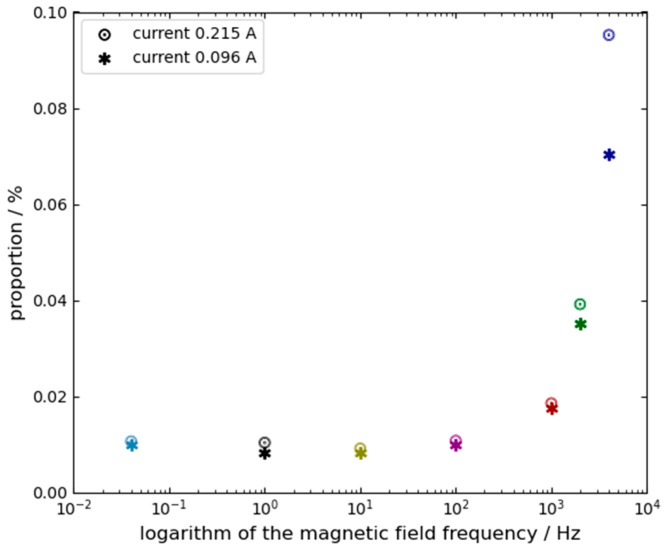
Variation in the optimal operating point position with magnetic field frequency.

The error E is extracted using a weighted sliding-window algorithm. It is important to note that since the magnetic field is modulated as a high-frequency square wave and the error is calculated as the difference between the positive and negative states, any static system bias or slow-drifting bias is naturally canceled out. Crucially, this high-frequency alternating modulation (up to 4 kHz) combined with the strict positive-negative differential calculation perfectly isolates the magnetic effects from potential alternative physical perturbations, such as thermal expansion of the gain medium or localized coil heating. Because thermal and structural dynamics possess macroscopic time constants that are orders of magnitude larger than our modulation period (e.g., 0.25 ms at 4 kHz), their slow-varying contributions are entirely subtracted out in Equation (8). Furthermore, visual and electrical monitoring confirmed that the minimal applied currents (0.016 A to 0.215 A) did not induce any observable changes in plasma brightness or DC discharge balance, strictly confining the observed error E to magneto-optic and electro dynamic interactions. Therefore, the “Average Error” presented in the results represents the specific sensitivity of the gyro to the magnetic field (Lorentz and Zeeman effects) rather than a shift in the underlying system bias. Experimental data are shown in Table 1. Based on these data, Figure 5 and Figure 6 present the average errors and the optimal operating point positions, respectively, as functions of the magnetic field frequency and amplitude. In both figures, the horizontal axis represents the frequency of the applied magnetic field on a logarithmic scale. The symbols ∗ and ☉ denote the magnetic fields generated by coil currents of 0.096 A and 0.215 A, respectively. The different colors in both figures are only for improving readability.

## 5. Data Analysis and Conclusions

### 5.1. Data Analysis

1. Based on the data in Table 1 and the trends shown in Figure 5 and Figure 6, several observations can be made: First, when the magnetic field frequency exceeds 100 Hz, the position of the optimal operating point and the average error are significantly affected by the frequency and amplitude of the magnetic field. The impact intensifies as either the frequency or the amplitude increases. Therefore, the position of the optimal operating point is decided by both the frequency and the amplitude of the magnetic field. Second, when the magnetic field frequency is lower than 100 Hz, the position of the optimal operating point becomes relatively insensitive to variations in the frequency and amplitude of magnetic field;
2. From Table 1, it can also be concluded that the frequency of the magnetic field remains a critical factor influencing the position of the operating point even under low-amplitude magnetic field.

### 5.2. Conclusions

1. A FFDLG does not have the universal “optimal operating point” that can ensure insensitivity to all external magnetic fields. In fact, the position of the optimal operating point is dynamic, shifting according to the frequency and amplitude of the magnetic field;
2. Consequently, it is challenging to take the optimal operating point to eliminate the FFDLG magnetic-induced error sin practical applications, which limits the practical utility of this approach. This is because environmental magnetic fields are unknown, so which optimal operating point should be chosen cannot be determined.

## 6. Primary Recognition of Physical Origin

To quantitatively describe the Lorentz force effect on the plasma and validate the experimental observables, we consider the dynamic equation of motion for charged particles within the gain region. When subjected to an alternating magnetic field $B=B_{0}\sin(\omega t)$, the ionized particles acquire an axial velocity component. The equation of motion for a particle with mass m and charge q is governed by $m\frac{dv}{dt}=q(E+v_{r}\times B)-\frac{mv}{\tau }$, where $v_{r}$ is the radial drift velocity and τ is the collision relaxation time.

Solving this for high-frequency steady-state conditions reveals a strong mass dependency. While light electrons ($m_{e}$) can track the alternating field synchronously, heavy neon ions ($m_{i}\gg m_{e}$) experience significant phase lag and inertial damping. This disparity creates a net macroscopic velocity imbalance Δv between the ionic lattice and the electron gas along the optical axis, which can be approximated as $\Delta v\approx \frac{qB_{0}}{m_{i}\omega }v_{r}$.

This flowing plasma acts as a moving medium, inducing a non-reciprocal frequency shift between the CW and CCW beams via the Fresnel drag effect. The resulting frequency shift Δf (which manifests as our observed magnetic error E) is quantitatively described by:

(10) $$\Delta f=\frac{2d}{\lambda L}(n^{2}-1)\Delta v$$

where d is the active length of the discharge plasma, λ is the resonant wavelength, L is the total cavity length, and n is the refractive index of the gain medium. Plugging the standard parameters of our FFDLG into this framework yields an expected frequency bias in the range of tens of Hz at typical excitation currents, which firmly aligns with the quantitative magnitude of the average errors recorded in our high-frequency trials (Table 1).

Under the assumption that the influences of the magnetic field on the FFDLG are solely governed by the Zeeman effect, the FFDLG possesses a unique “optimal operating point”, where it is insensitive to all external magnetic fields. However, experimental results contradict this. In reality, external magnetic fields also interact with the plasma motion within the gain region via the Lorentz force. The specific mechanism is as follows:

External magnetic fields change the velocity and direction of the plasma in the gain region of the gyroscope via the Lorentz force. Specifically, the ionized particles (electrons and ions) in the discharge tube acquire a velocity component along the optical axis when subjected to a magnetic field. This induced plasma flow acts as a moving medium, which gives rise to the Fresnel drag effect (also known as the Fizeau effect). According to the theory of Fresnel drag, the phase velocity of light is modified by the velocity of the medium through which it propagates. In an RLG, the CW and CCW beams experience different phase shifts due to this flow, creating a non-reciprocal frequency shift.

This drag-induced imbalance contributes significantly to the total gyro bias. All changes to the plasma motion will result in both Doppler shifts in light atoms and changes to the effective optical path length through Fresnel drag, leading to the drift of the gyroscope. This physical phenomenon can be explained qualitatively by the disparate response times of electrons and ions. At higher magnetic field frequencies, the motion of electrons can track the alternating field synchronously; however, heavier ionized atoms(such as Ne^20^ and Ne^22^) fail to synchronize due to their greater inertia, leading to larger induced errors. Conversely, at lower frequencies, both electrons and ions can respond synchronously to the magnetic field variations, thereby minimizing the resultant error. Thus, the experimental “optimal operating point” is actually a position where the combined errors from both the Zeeman effect and the Lorentz force are minimized.

This model accounts for two phenomena observed in the FFDLG experiments. First, while conventional theory suggests that transverse magnetic field have negligible effects on the gain region, they in fact cause output drift by perturbing plasma motion through the Lorentz force. Second, it explains the significant variations in drift observed between different working states of the FFDLG, as reported in Ref. [19].

## 7. Further Experimental Validation

“To validate the aforementioned theoretical framework and to strictly isolate the Lorentz-force-driven plasma motion from standard Zeeman splitting, supplementary experiments were designed as strict control trials. Following the experimental procedure detailed in Section 4, the gyroscope was operated in an inverted distinct state. In this methodology, the near-static test conducted at 0.04 Hz (with 0.215 A) serves as the baseline control experiment. At this ultra-low frequency, both heavy ions and electrons can respond synchronously to the magnetic field, thereby minimizing the Lorentz-induced velocity imbalance (Δv≈0). Consequently, the Zeeman effect dictates the error. Conversely, the high-frequency tests at 4 kHz serve as the primary experimental group, where the Lorentz force dominates the plasma dynamics. The isolated effects were measured and are illustrated in Table 2.

## 8. Analysis Data Again

Table 2 demonstrates that in the alternative operating state, the optimal frequency stabilization point continues to vary with the magnetic field frequency and amplitude, which is consistent with the conclusions drawn previously.

According to the theoretical model in Equation (3), if the Zeeman effect were the dominant influence, the average error and the direction of the optimal operating point should invert when switching between the two operating states. However, a comparison of Table 1 and Table 2 reveals that this prediction only aligns with experimental data at 0.04 Hz. At 4 kHz, the experimental results contradict this theoretical expectation. This discrepancy can be explained by the following mechanisms:

1. External magnetic fields directly perturb the plasma motion within the gain region. Unlike the Zeeman effect, whose induced error reverses direction when the gyroscope’s operating state is switched, the error caused by Lorentz-force-driven plasma motion maintains the same direction regardless of the operating state;
2. At high frequencies, the error induced by magnetic fields via the Lorentz force dominates, while the relative contribution of the Zeeman effect is significantly smaller. Consequently, the main magnetic field effect error is the error induced by the plasma motion by Lorentz force, the optimal operating point’s direction remains the same across different operating states because it is primarily governed by the Lorentz force. At low frequencies, the Lorentz-force-induced error is reduced, allowing the Zeeman effect to become the primary source of error. Therefore, the main magnetic field effect error is the Zeeman effect error, the optimal operating point shifts in opposite directions when the gyroscope state is switched, consistent with Zeeman theory;
3. Under a static magnetic field, the Lorentz-force-induced error manifests as a constant or quasi-static bias. This bias is often inadvertently merged with the Zeeman error, making it difficult to distinguish. This also confirm that the magnetic effects in the FFDLG are not limited to the Zeeman effect alone.

## 9. Conclusions

### 9.1. Conclusions of This Paper

On the basis of the experiments and analyses presented above, the following conclusions are drawn:

1. The FFDLG does not possess a universal “optimal operating point” that renders it insensitive to external magnetic fields across all frequencies and amplitudes. Instead, the position of this optimal point is dynamic, varying significantly as a function of the magnetic field’s frequency and amplitude;
2. Similar conclusions apply to Mechanically Dithered Ring Laser Gyros (MDRLG). In these devices, magnetic fields also alter plasma motion via the Lorentz force, leading to Doppler shifts and additional drift. This effect has likely been overlooked in previous laboratory tests because typical sampling intervals (e.g., 1 s or 10 s) tend to average out alternating magnetic field errors into a quasi-static bias. Furthermore, since high-frequency magnetic field testing is uncommon, the Lorentz-force-induced error under static fields is often inadvertently conflated with the Zeeman effect error, remaining largely undetected;
3. The sensitivity of plasma motion to external magnetic fields restricts the practical utility of optimal operating point control schemes. This limitation is particularly pronounced in environments with high-frequency magnetic interference, where alternating magnetic field errors intensify. In such cases, a selected optimal operating point may only be effective for a specific high-frequency component rather than providing broad-spectrum protection against varying magnetic interference.

### 9.2. Some Discussions About “The Optimal Frequency Stabilization Operating Point” in Practical Application

Based on the findings of this study, the following scenarios for practical application are discussed:

1. If the characteristics of external magnetic fields are well-characterized in a specific application, the operating point can be dynamically adjusted to suppress magnetic-induced errors. In such cases, the “optimal frequency stabilization operating point” remains a viable approach;
2. For applications with low output frequency requirements, a low-frequency magnetic field can be applied to modulate the operating point. This effectively mitigates errors induced by low-frequency external fields, making the optimal operating point control applicable;
3. In high-frequency applications, such as inertial navigation systems and attitude control, the optimal operating point can be determined using a low-frequency magnetic field prior to system installation or during the pre-deployment startup phase. Once identified, the gyroscope can be maintained at this stabilized point throughout its operation to reduce low-frequency magnetic interference;
4. Robust magnetic shielding remains the fundamental solution for minimizing magnetic-induced errors. The utility of the “optimal operating point” method is inherently limited on its own. However, when integrated with high-performance shielding, the benefits are compounded: the operating point control handles residual low-frequency deviations, while the shielding effectively attenuates high-frequency interference. This combined approach can reduce magnetic-induced drift to negligible levels.

Through experimental analysis and theoretical investigation, this paper concludes that Lorentz-force-induced plasma motion and the resulting Fresnel drag effect are significant sources of magnetic error in FFDLGs. This finding refutes the traditional assumption that an FFDLG possesses a unique, universal “optimal operating point” insensitive to all magnetic interference. The research presented here enriches the theory of magnetic effects in FFDLGs and provides a clearer technical direction for error suppression. While the current explanation provides a coherent physical model for the experimental results, the underlying fundamental processes warrant further quantitative investigation. To the best of our knowledge, this study represents the first time the influence of the Lorentz force on plasma dynamics has been investigated as an error source in RLG.

## Figures and Tables

**Table 1:** Experiment data for magnetic-induced average errors and optimal operating point positions under different magnetic field parameters.

Current amplitude is 0.215 A							
Current frequency	4 KHz	2 KHz	1 KHz	100 Hz	10 Hz	1 Hz	0.04 Hz
Average error	22.17	13.62	7.44	5.01	4.22	4.53	4.72
Percent	9.52%	3.92%	1.86%	1.08%	0.92%	1.04%	1.07%
Current amplitude is 0.096 A							
Current frequency	4 KHz	2 KHz	1 KHz	100 Hz	10 Hz	1 Hz	0.04 Hz
Average error	10.72	6.43	3.53	2.17	1.65	1.66	2.07
Percent	7.03%	3.51%	1.77%	1.01%	0.84%	0.84%	1.00%
Current amplitude is 0.016 A							
Current frequency	4 KHz	2 KHz	1 KHz	100 Hz	10 Hz	1 Hz	0.04 Hz
Average error	1.89	---	---	---	---	---	0.302
Percent	5.80%	---	---	---	---	---	0.85%

**Table 2:** Experimental validation data for magnetic-induced average errors and optimal operating point positions under different magnetic field parameters.

Current amplitude is 0.215 A		
Current frequency	4 KHz	0.04 Hz
Average error	14.54	−10.095
Percent	5.60%	−1.78%
Current amplitude is 0.096 A		
Current frequency	4 KHz	---
Average error	6.25	---
Percent	3.74%	---

## Data Availability

The original contributions presented in this study are included in the article. Further inquiries can be directed to the corresponding author.
